# Changes in mental health across the COVID-19 pandemic for local and international university students in Australia: a cohort study

**DOI:** 10.1186/s40359-023-01075-9

**Published:** 2023-02-28

**Authors:** Melissa A. Russell, Nicola Reavley, Ian Williams, Wenjing Li, Laura Tarzia, Patty Chondros, Lena Sanci

**Affiliations:** 1 Centre of Epidemiology and Biostatistics, Melbourne School of Population and Global Health, University of Melbourne, Melbourne, Australia; 2 Centre of Mental Health, Melbourne School of Population and Global Health, University of Melbourne, Melbourne, Australia; 3 Department of General Practice, Melbourne Medical School, University of Melbourne, Melbourne, Australia

**Keywords:** Depression, Anxiety, University students, Pandemic, Social support, International student, Social support

## Abstract

**Purpose:**

Previous research has indicated that university students experienced substantial mental health issues during the global COVID-19 pandemic, but few studies have considered changes relative to pre-pandemic levels across population groups. Hence, the aim of this study was to compare changes in mental health and associated stressors across the pandemic for international and local university students studying in Australia.

**Methods:**

In a cohort of 4407 university students, we assessed depression (Patient Health Questionnaire 2), anxiety (Generalized Anxiety Disorder-2), social support (Medical Outcomes Study—Social Support Survey), inability to afford food, fear of partner, and experiences of discrimination, both pre-pandemic (April–May 2019) and during the pandemic (September–October 2020). Change in prevalence between local and international students were estimated with logistic regression, adjusting for baseline factors.

**Results:**

Compared to local students, international students experienced an increase in probable major depression (odds ratio (OR) 1.43, 95% Confidence Interval (CI) 1.23, 1.66), low social support (OR 2.63, 95% CI 2.23, 3.11), inability to afford food (OR 5.21, 95% CI 3.97, 6.83) race-based discrimination (OR 2.21, 95% CI 1.82, 2.68) and fear of partner (OR 3.46, 95% CI 2.26, 5.13). Interaction analyses indicated that these issues were more likely to be experienced by students living outside their country of origin, inclusive of international students based in Australia (depression *p* value interaction term 0.02).

**Conclusion:**

The pandemic had a substantial negative impact on international students, particularly those living outside of their country of origin during the pandemic. The inequalities exacerbated by the pandemic were present prior to the pandemic and are likely to continue post-pandemic without action. Interventions to build the supports for international students need to be urgently explored.

**Supplementary Information:**

The online version contains supplementary material available at 10.1186/s40359-023-01075-9.

## Introduction

It is estimated that 34% of the global young adult population enrolls in tertiary study within five years of completing secondary school [1]. This proportion is predicted to increase in coming years [1]. Enrolment in university typically occurs during the same life stage that mental health issues commonly arise [2], hence universities are responsible for young people at a time of high mental health risk. To add to the challenges for many young adults entering university, the number of young people travelling internationally for tertiary study has been increasing in recent years, doubling from 2007 to 2019 [3]. While the United States and United Kingdom attract the largest number of international students, increases have been observed in many countries.

Prior to 2020, the mental health and general wellbeing of university students was a growing area of concern worldwide [4–9]. Numerous studies highlighted the high prevalence of mental health issues in this population [4–8]. Other stressors, such as financial stress, relationship issues and changing social support structures, have also been observed for university students, and have been found to be associated with mental health issues [6–8]. For students travelling abroad for tertiary study (international students) there exists a unique set of additional stressors, such as acculturative stress, potential discrimination, and stress related to language proficiency concerns [10–12]. Additionally, international students, who are living away from their country of origin, are separated from their long-term social supports [13]. These factors have been found to contribute to mental health issues in international students [10–13].

With the onset of the global COVID-19 pandemic in 2020 many countries enacted restrictions to curb the spread of the disease. Subsequently, many cross-sectional studies reported a high prevalence of mental health issues in university students during the pandemic period [14–17]. Reviews found that 39.4% and 31.2% of university students worldwide experienced anxiety and depression, respectively, during the COVID-19 pandemic [18]. Whilst these studies demonstrated relatively high levels of mental health issues, only a limited number of longitudinal studies had the capacity to assess *changes* in university student mental health over the COVID-19 pandemic period [19]. One of the largest longitudinal studies, a cohort study of 500 students in the UK, found that the prevalence of “higher than normal” depression levels was double that of pre-pandemic levels [20]. Whilst these few longitudinal studies pointed to potential increases in prevalence of mental health issues due to the pandemic, the sample sizes were relatively small and there was no examination of specific population group outcomes (i.e., comparison across local and international student groups).

As in the pre-pandemic period, international students appeared to face additional stressors during the COVID-19 pandemic, compared to their local counterparts. In a cross-sectional comparison of 606 local and 181 international university students in Australia, international students were more likely than local students to report seeking support from university services, experience anxiety about the future, and encounter financial stress [21]. However, results from this study were obtained from a cross-sectional study, hence pre-pandemic comparison was not available.

A further issue that remains unexplored is the comparison in psychosocial outcomes for students living across different pandemic countries of residence. Depending on the nature and timing of COVID-19 restrictions, some international students remained in their country of study during the pandemic, whilst others chose to return to their country of origin or were forced to remain in their country of origin due to restrictions on travel. It can be hypothesized that international students living in their country of origin were separated from their university but had the benefit of long-term social supports. The opposite would be true for international students living in their country of study during the pandemic; they would be separated from family support [13, 22] but closer to their studies. Another influential factor in this comparison would be that countries differed in their levels of restrictions and COVID-19 prevalence; both factors have been found to impact resident’s mental health [23, 24]. These differences would potentially lead to different sets of stressors experienced based on student country of residence, which has not been previously investigated.

To provide a clearer picture of the impact of the pandemic on university students’ wellbeing, we compared mental health and associated stressors for international and local students across the pre-pandemic and pandemic periods, and considered the potential impact of students’ pandemic country of residence. This information could be utilized by universities and mental health services to inform potential support and recovery strategies, and the targeting of such strategies to different population groups. Hence, the aims of this study were to:

1. Compare the change in prevalence of mental health issues and associated stressors (racism, food security, social support, and fear of partner) from pre-pandemic to during the pandemic for local and international university students
2. Explore the modifying effect of pandemic country of residence on the change in prevalence of mental health issues and associated stressors (racism, food security, social support, and fear of partner)
3. Report the prevalence of pandemic stressors in both local and international university students during the pandemic.

## Methods

The “Towards a Health Promoting University” cohort study was undertaken at a single tertiary education institution in Melbourne, Australia, across two waves: April/May, 2019 (Wave 1) and September/October, 2020 (Wave 2). All undergraduate, postgraduate, and graduate research students aged 18+  and enrolled at the University were invited via direct email to participate in the original 20-min online health and wellbeing survey in 2019. Of the 56,375 students invited, 14,880 students participated in Wave 1 (for full details of methods see Sanci and colleagues [25]). For Wave 2, all students who responded to the Wave 1 survey and remained enrolled at the university in 2020 were contacted again and invited to participate in a shorter, five-minute Wave 2 online survey. At the time of the Wave 2 data collection the city of Melbourne was in strict lockdown (e.g., from 2 August to 28 October 2020 residents could leave the house only for exercise, caring, seeking medical care, or groceries). Hence university teaching was being delivered online rather than in-person. Ethics approval for this study was received from the participating University (Medicine and Dentistry Human Ethics Sub-Committee approval: 2,057,722) and informed consent from all participants was received.

### Data collection

#### Demographics

The following demographic data collected at Wave 1 are reported in this study:

- *Age* (continuous)
- *Gender* (male, female, non-binary)
- *Citizenship status* was categorized as local or international student (independent variable). An international student was defined as someone holding an Australian temporary resident student visa or bridging visa who had come to Australia to study. A local student was defined as someone who was an Australian citizen or permanent resident.
- *Course level*, categorized as undergraduate, post-graduate, research higher degree (PhD, Masters by Research (RhD)).

At Wave 2 additional demographic questions regarding place of residence and living arrangements were included:

- *Pandemic country of residence* was assessed by asking which country students were residing in (independent variable). Country of residence was categorized into ‘living overseas’ and ‘living in Australia’.
- *Pandemic living arrangements* was assessed by asking students who they were currently living with. Data were categorized into living with family (living with parents, dependent children only, other relatives), living with partner (with and without others) living with others (friends/housemates, residential college) or living alone.

#### Dependent variables repeated across Wave 1 and Wave 2

- *Depression* was measured using the Patient Health Questionnaire 2 (PHQ2) [26]. The 2-item PHQ-2 comprises the items ‘Over the last two weeks how often have you had little interest or pleasure in doing things’ and ‘Over the last two weeks how often have you felt down, depressed, or hopeless’. The response options were ‘not at all, ‘several days’ ‘more than half the days’ and ‘nearly every day’. The two items were summed to obtain a scale score, with a recommended cut-off of three or more indicating probable major depression. The PHQ-2 has been found to be a valid indicator of major depression in university students in China, with a sensitivity and specificity of 81% and 96%, respectively [27].
- *Anxiety* was measured using the Generalized Anxiety Scale 2 (GAD-2) [28]. The 2-item GAD-2 comprises the items ‘Over the last 2 weeks, how often have you been bothered by feeling nervous, anxious or on edge’ and ‘Over the last 2 weeks, how often have you not being able to stop or control worrying’. Response options were the same as those in the PHQ-2. Scale scores was the sum of these items, with a recommended cut-off of three or more indicating probable generalized anxiety disorder [29]. The GAD-2 has been found to be a valid representation of the GAD-7, with a sensitivity of 84% to 87% and specificity of 93% to 95%, for men and women, respectively [30].
- *Social support* was measured using the MOS Social Support Survey (MOS-SSS-6) [31]. The MOS-SSS-6 consists of six items gauging the presence of social support. Example items include ‘Someone to share private worries and fears’ and ‘Help if you are confined to bed’. A five-point scale is used to indicate how often these social supports are available (ranging from ‘none of the time’ to ‘all of the time’). The sum of the items ranged between 6 and 30, and to aid interpretation was dichotomized as “less than 18” vs “18 or more”, with higher total score indicating greater perceived support. The MOS-SSS-6 has been found to have a Cronbach’s alpha of 0.70 and reliability of 0.89 [31].
- *Food security* was gauged with the item ‘In the last 12 months were there any times that you ran out of food and could not afford to buy more’, with response options comprising ‘yes’, ‘no’, and ‘unsure’. ‘Unsure’ and ‘no’ were collapsed for this analysis. This item was based upon a previous study of food insecurity [32].
- *Experiences of discrimination based on race, ethnicity and gender* was gauged with the item ‘Have you experienced discrimination in the wider community on the basis of…’. Respondents were presented with a range of response options including ‘race’, ‘ethnicity’ and ‘gender’. These items were adapted from a previous study [33].

#### Dependent variables collected in Wave 2 only (pandemic stressors)

- *Fear of partner during the pandemic* was gauged in a newly devised item ‘During the COVID-19 pandemic, have you been afraid of a partner’. Response options were ‘yes’, ‘no’ and ‘I don’t have a partner’. A second item, ‘What is your level of fear’ was posed to people who answered ‘yes’ to the first question. Response options were ‘my level of fear has been the same as prior to the COVID-19 pandemic’, ‘my level of fear has increased with the COVID-19 pandemic’, and ‘my level of fear has decreased with the COVID-19 pandemic’. Whilst fear of partner items were also included in Wave 1, the items were modified in Wave 2 due to survey length limitations and to capture the context of the pandemic more precisely.
- *Pandemic stressors* Possible stressors experienced by students during the pandemic were assessed with a new item. The question posed was ‘During the COVID-19 pandemic have any of the following areas of your life changed’. Items included were ‘my physical health’, homesickness’, ‘access to mental health services’, ‘access to general health services’, ‘coping with study’, ‘confidence in future job prospects’, ‘racial discrimination’, ‘connection to the university’, ‘connection to university peers’, ‘connection with friends’, ‘relationship with my partner’, ‘relationship with my family’, ‘financial position’, ‘access to paid work’, ‘suitable accommodation/living arrangements’, and ‘finding a suitable place to study’. A five-point rating scale was used (‘much worse than before’, ‘somewhat worse than before’, ‘unchanged’, ‘better than before’, and ‘much better than before’). In this report the two ‘worse’ options were combined and the two ‘better’ options were combined. This item was developed by the project team based on a qualitative analysis of student and staff reports of the issues being experienced by students at the time of the pandemic.

### Analysis

Means, standard deviations (SD), proportions with confidence intervals were utilized to describe the prevalence of mental health issues and associated stressors in the pre-pandemic period (Wave 1) and during pandemic period (Wave 2). Descriptive statistics were reported by citizenship status (local and international students). The COVID-19 pandemic stressors were reported for Wave 2 only.

Multifactorial logistic regression was utilized to assess the association between citizenship status (international students versus local student) and the prevalence of depression, anxiety, and other stressors at Wave 2. Analyses included adjustment for age, gender, and baseline levels of the relevant factor. Tests for interaction was used to investigate if the association between citizenship status and each outcome was modified by pandemic country of residence, and living arrangements, respectively. To investigate (1) the effect of loss to follow-up at Wave 2, and (2) the effect of missing data at baseline, an inverse proportional weighting (IPW) was applied to regression analyses (methodology included in the Additional file 1) [34].

Initial power analysis for Wave 1 indicated that 720 students were required to detect a 0.2 standard deviation difference in the mean PHQ score of depressive symptoms across a dichotomous exposure with 80% power and a 5% two sided-alpha level. As these numbers were well exceeded by the recruitment strategies the power analyses are not reported in detail.

## Results

### Participants

Of the 14,880 students who completed the Wave 1 survey, 9011 students were still enrolled at Wave 2, approximately 18 months later. Relative to students in the original cohort, those students still enrolled by Wave 2 tended to be younger, and in a three-year undergraduate degree, rather than a shorter post-graduate degree (Table 1). Of the 9011 students who participated in Wave 1 and remained enrolled during Wave 2, a total of 4407 students responded to the Wave 2 questionnaire (Table 1). At Wave 1, those responding in Wave 2 were a similar age to all participants in Wave 1, were slightly more likely to be local students (71.8% at Wave 2 compared to 67.3% at Wave 1), and more likely to be female (68.3% at Wave 2 compared to 63.3% at Wave 1).

**Table 1:** Demographic characteristics of participants pre-pandemic (Wave 1)

	Wave 1 population (n = 50,930)	Wave 1 participants (n = 14,880)	Wave 1 students continuing at university across 2019 and 2020 (n = 9011)	Students who responded to the Wave 2 survey (n = 4407)	Local students who responded to the Wave 2 survey (3162)	International students who responded to the Wave 2 survey (1245)
*WAVE 1*						
Mean age (SD)	24.8	24.2 (6.9)	23.1 (6.4)	23.4 (6.8)	23.4 (7.5)	23.3 (4.5)
Median age (IQR)		22 (20, 26)	21 (19, 24)	21 (19, 25)	21 (19, 24)	22 (20, 26)
Gender (% of total)						
Female	(56.7)	9432 (63.9)	5662 (63.3)	2991 (68.3)	2166 (68.9)	825 (66.9)
Male	(43.1)	5251 (35.6)	3239 (36.2)	1361 (31.1)	954 (30.3)	407 (33.0)
Self-described	(0.1)	77 (0.5)	43 (0.5)	28 (0.6)	26 (0.8)	2 (0.2)
Missing		120	67	27	16	11
Citizenship status (% of total)						
Local	(62.3)	9412 (63.3)	6064 (67.3)	3162 (71.8)	N/A	N/A
International	(43.1)	5468 (36.8)	2947 (32.7)	1245 (28.3)	N/A	N/A
Missing		0	0	0		
Study level (% of total)						
Undergraduate		6830 (46.70)	4954 (55.9)	2329 (53.4)	1772 (56.5)	557(45.4)
Masters (coursework)		6001 (41.03)	2781 (31.4)	1403 (32.2)	985 (31.4)	418 (34.0)
Other postgraduate		346 (2.37)	164 (1.9)	86 (2.0)	70 (2.2)	16 (1.3)
Graduate research		1448 (9.90)	959 (10.6)	544 (12.5)	307 (9.8)	237 (19.3)
Missing		225 (1.71)	153	45 (1.0)	28	17
*WAVE 2*						
Pandemic Country of residence (% of total)						
Overseas		N/A	N/A	232 (5.3)	35 (1.1)	197 (15.8)
Australia		N/A	N/A	4174 (94.7)	3127 (98.9)	1047 (84.2)
Missing				1	0	1
Living circumstances (% of total)						
With family					1690 (53.5)	246 (19.8)
With partner					587 (18.6)	208 (16.7)
With others					640 (20.3)	467 (37.5)
Alone					244 (7.7)	324 (26.0)
Missing				1	1	0

*SD* Standard deviation *IQR* Interquartile range *N/A* Not applicable

The average age of participants in the present study at Wave 1 was 23.4 years (standard deviation (SD) 6.8) and 68.3% were female, 31.1% were male and 0.6% were non-binary. The characteristics of participating local and international students were similar in terms of age and gender (Table 1). Only 1.1% of local students were living overseas during Wave 2 whilst 15.8% of international students were living overseas. Additionally, more international students were living alone (26.0%), compared to local students (7.7%).

### Change in mental health and related stressor prevalence across local and international students

From the pre-pandemic period (Wave 1) to during the pandemic (Wave 2) the percentage of students overall with probable major depression increased from 21.7% (95% confidence interval (CI) 20.4%, 23.0%) to 36.7% (95% CI 35.3%, 38.1%). A similar increase was observed in probable anxiety levels from pre-pandemic to Wave 2 (Table 2). During the pandemic, international students were at much greater risk of an increase in probable major depression than local students, with the odds for international students almost 1.5 times that of local students (OR: 1.43 (95% CI 1.23, 1.66). The difference between international students and local students was not as stark for probable anxiety (Table 2). There were no material differences observed when the data was weighted for non-participation (Additional file 1: Table S1).

**Table 2:** The prevalence and odds ratios of mental health issues and associated stressors across local and international students

	All students (n = 4407)		Local students (3162)		International students (1245)		Adjusted odds ratio of outcome for international students compared to local student (n = 4407)*
	Wave 1	Wave 2	Wave 1	Wave 2	Wave 1	Wave 2	
Percentage with probable major depression (PHQ2 ≥ 3) (95% CI)	21.7 (20.4, 23.0)	36.7 (35.3, 38.1)	21.7 (20.4, 23.0)	34.4 (32.8, 36.1)	22.8 (20.4, 25.4)	42.6 (40.0, 45.4)	1.43 (1.23, 1.66) *p* < 0.001
Missing	456	66	311	38	145	28	535
Percentage with probable anxiety (GAD2 ≥ 3) (95% CI)	32.3 (30.1, 33.4)	47.0 (45.6, 48.5)	34.1 (32.4, 35.9)	47.0 (45.3, 48.8)	27.7 (25.1, 30.4)	46.9 (44.1, 49.7)	1.09 (0.94, 1.27) *p* = 0.26
Missing	527	55	356	28	171	27	591
Percentage reporting low social support (MOS-SSS-6 < 18) (95% CI)	44.9 (43.3, 46.5)	46.3 (44.9, 47.8)	37.6 (35.9, 39.4)	37.9 (36.2, 39.6)	63.9 (61.0, 66.7)	68.0 (65.4, 70.7)	2.64 (2.23, 3.12) *p* < 0.001
Missing	469	66	318	38	151	28	612
Percentage unable to afford food (95% CI)	9.4 (8.6, 10.4)	7.7 (6.9, 8.5)	10.0 (8.9, 11.2)	4.3 (3.7, 5.1)	7.9 (6.4, 9.6)	16.3 (14.3, 18.5)	5.21 (3.97, 6.83) *p* < 0.001
Missing	408	18	278	12	130	6	447
Percentage experiencing discrimination (95% CI)							
Based on race	22.0 (20.8, 23.4)	22.6 (21.3, 23.8)	17.6 (16.2, 19.1)	17.4 (16.1, 18.7)	33.6 (30.8, 36.5)	35.9 (33.2, 38.7)	2.21 (1.82, 2.68) *p* < 0.0001
Based on ethnicity	16.6 (15.4, 17.8)	17.0 (15.9, 18.2)	15.3 (14.0, 16.7)	15.2 (13.9, 16.5)	20.2 (17.9, 22.7)	21.9 (19.6, 24.3)	1.58 (1.29, 1.94) *p* < 0.0001
Based on gender	28.1 (26.7, 29.6)	24.9 (23.7, 26.3)	33.3 (31.6, 35.0)	29.5 (27.9, 31.1)	14.7 (12.7, 16.9)	13.2 (11.4, 15.3)	0.51 (0.41, 0.54) *p* < 0.0001
Missing	537	91	363	54	174	37	625
Percentage reporting fear of partner during the pandemic (95% CI)		3.4 (2.7, 4.1)		2.1 (1.6, 2.9)		7.1 (5.4, 9.3)	3.46 (2.26, 5.13) *p* < 0.001
No partner/missing		2640		1185		582	1802
Percentage reporting increased fear of partner during the pandemic (95% CI)		82.0 (72.5, 88.8)		78.6 (63.2, 88.7)		85.1 (71.5, 92.9)	1.76 (0.54, 5.75) *p* = 0.350
No fear/no partner/missing		4334		3120		1245	4318

*PHQ2* Patient Health questionnaire-2 item, *GAD2* Generalized anxiety disorder questionnaire-2 item, *CI* Confidence interval

*Adjusted for age, gender and baseline levels of the relevant factor

When comparing the prevalence of different stressors across all students, there was little difference between Wave 1 and Wave 2 (Table 2). There was a small drop in the number of students reporting inability to afford food and a small increase in the number of students reporting low social support (Table 2). However, these whole cohort changes over time masked the difference in experiences of local and international students.

International students were more likely than local students to report lower social support during the pandemic (OR 2.64 (95% CI 2.23, 3.12). This trend was on the back of international students already reporting substantially lower levels of social support compared to local student’s pre-pandemic (Table 2). International students experienced a fivefold increase in the odds of being unable to afford food during the pandemic compared to local students (OR 5.21 (95% CI 3.97, 6.83). International students also reported increases in race-based discrimination (OR 2.21, 95% CI 1.82, 2.68) and discrimination based on ethnicity (OR 1.58, 95% CI 1.29, 1.94) during the pandemic, compared to local students. In comparison, there was a decrease in reporting of gender-based discrimination for all students.

Fear of partner during the COVID-19 pandemic was reported by 3.4% (95% CI 2.7%, 4.1%) of students overall. Most of these students (82.0%) reported increased levels of fear from pre-pandemic levels. The odds of reporting fear of partner during the pandemic were 3.46 times (95% CI 2.6, 5.13) higher for international students compared to local students (Table 2).

All analyses reported above were repeated with the data weighted for non-participation to account for missing data (Additional file 1: Table S1) and with outcomes treated as continuous variables (Additional file 1: Table S2); no material differences were observed from those reported above.

### Effect modification of pandemic country of residence on the association between local/international student status and outcomes

**Fig. 1:**
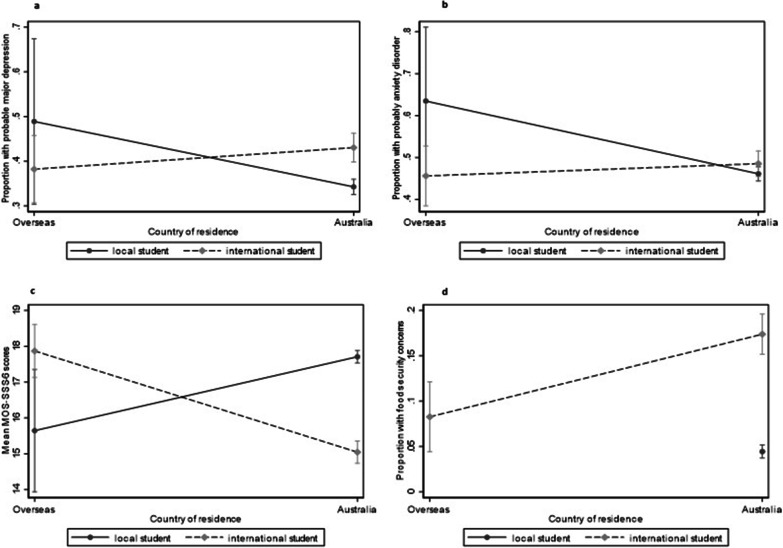
The modifying effect of pandemic country of residence on the association between citizenship status (local/international student) and **a** probable depression, **b** probable anxiety, **c** social support, and **d** food security

Several statistical interactions were observed between country of residence and student citizenship status (Fig. 1a–d, Additional file 1: Table S3). International students living in Australia were more likely to experience probable major depression than international students living in their country of origin/overseas during the pandemic, and local students displaced from Australia and living overseas similarly were more likely to experience probable major depression (interaction term *p* value = 0.02). (Fig. 1a). Similar results were observed for probable anxiety (*p* value for interaction term 0.05) and social support (interaction term *p* value = 0.01). International students living in Australia were more likely to experience food security concerns (17.9%, 95% CI 15.7, 20.4) during the pandemic than international students living overseas (7.7%, 95% CI 0.6, 1.2) and local students living in Australia (4.4%, 3.7%, 5.1%), however statistical interactions were not able to be explored due to the low number of local students living overseas experiencing food security issues.

No modifying effect of living arrangements (with family, friends/other, alone) was observed on the association between citizenship status and outcomes.

### Pandemic stressors

Students reported that several issues worsened for them during the pandemic. The most prevalent of these included worsening connection to university peers (82.5%), connection to the university (80.1%), connection to friends (75.8%), concerns regarding future job prospects (72.0%) and mental health (70.8%) (Table 3). The issues that more students reported as improving more than worsening were relationship with family (27.5%) and relationship with partner (27.7%) (Additional file 1: Table S4).

**Table 3:** Self-reported impact of COVID-19 across local and international students overall and by pandemic country of residence for international students at Wave 2

	Number of students who reported the issue to be worse than before the pandemic n (%)				
Pandemic issue	All participants (n = 4407)	Local students (n = 3096)	International students (n = 1198)	International students living overseas (n = 197)	International students living in Australia (n = 1047)
*Health and wellbeing*					
My mental health	3041 (70.8)	2181 (70.5)	860 (71.8)	120 (64.2)	739 (72.7)
My physical health	2333 (54.2)	1642 (53.0)	691 (57.4)	90 (48.1)	601 (59.0)
Homesickness	1535 (47.0)	826 (39.2)	709 (61.0)	53 (28.3)	656 (64.4)
Access to general health service	1396 (34.0)	1074 (36.1)	322 (28.4)	41 (21.9)	281 (27.6)
Access to mental health service	917 (24.2)	685 (25.4)	232 (21.3)	41 (21.9)	191 (18.8)
Racial discrimination	666 (24.0)	270 (15.5)	396 (38.3)	63 (33.7)	333 (32.7)
*Study*					
Connection to the University	3441 (80.1)	2525 (81.6)	916 (76.3)	155 (83.8)	761 (74.8)
Coping with study	3008 (70.0)	2113 (68.3)	895 (74.6)	146 (78.1)	1018 (73.6)
Finding a suitable place to study	2606 (61.9)	1770 (58.6)	836 (70.6)	133 (72.3)	703 (69.2)
*Connections with others*					
Connection to university peers	3540 (82.5)	2586 (83.7)	954 (79.6)	157 (84.9)	797 (78.4)
Connection with friends	3255 (75.8)	2393 (77.5)	862 (71.7)	126 (68.1)	736 (72.4)
Relationship with my partner	585 (22.9)	412 (22.7)	173 (23.5)	27 (14.7)	146 (14.4)
Relationship with my family	904 (21.1)	688 (22.3)	216 (18.0)	26 (15.1)	190 (18.7)
*Finance and housing*					
Confidence in future job prospects	3062 (72.0)	2088 (68.2)	974 (82.9)	152 (82.2)	822 (81.5)
Access to paid work	2349 (60.0)	1590 (55.4)	759 (72.9)	119 (64.7)	640 (63.0)
Financial position	1833 (43.4)	1109 (36.5)	724 (61.3)	113 (61.4)	611 (60.1)
Finding suitable accommodation	674 (19.4)	366 (15.3)	308 (28.7)	61 (33.2)	247 (24.3)

When comparing between local and international students there were some differences (Table 3). International students were much more likely to report a worsening of racial discrimination (38.3%) during the COVID-19 pandemic. Additionally, international students were more likely than local students to report worsening confidence in future job prospects, concerns with access to paid work, homesickness, and a worsening financial situation (Table 3).

Local students reported worsening access to general health and mental health services and worsening connection with the university, friends, and family at slightly higher rates than international students (Table 3).

When comparing the percentage of worsening issues amongst international students by pandemic country of residence, those students living in Australia (i.e., away from their country of origin) were more likely to report worsening mental health, physical health, and homesickness during the pandemic than international students living overseas (i.e., country of origin) (Table 3). International students living overseas were more likely to report worsening connection with university peers and the university during the pandemic and more difficulty finding suitable accommodation (Table 3).

## Discussion

Findings indicate that, compared to local students, international students experienced a substantially greater deterioration in mental health and social support during the pandemic. They also experienced food security issues and discrimination based on race and ethnicity at higher rates than local students. Of note, during the pandemic almost 70% of international students reported experiencing low social support, and 16% reported being unable to afford food in the previous months. When considering pandemic country of residence, the international students living in their country of study experienced worse mental health, lower social support, and a much greater likelihood of experiencing food security issues compared to those living overseas during the pandemic. The issues of poorer mental health and social support were mirrored for local students living overseas, although the numbers available for comparison were small. Local students too experienced a wide range of mental health and wellbeing impacts with the pandemic and associated restrictions.

Our findings support the theory that many issues compounded each other to lead to worsening mental health for university students, and particularly international students [35]. The restrictions in Australia during the pandemic impacted the social and psychological wellbeing of many people living there at the time [36]. It has also been observed that those experiencing greater mental health inequalities were most likely to be affected by the pandemic [36]; financial issues, lack of social support and racism having all been found to impact mental health [7, 37, 38]. Hence with the pre-pandemic issues already experienced by international students, the heightened impact from the pandemic is not surprising.

Some of the students in the present study may have been affected by the increase in pandemic related anti-Asian sentiment and racism that was observed globally [39, 40]. Australia has a relatively high proportion of students from Asian countries, and our results showed that reporting of race-based and ethnicity-based discrimination increased with the pandemic, particularly for international students. By contrast, gender-based discrimination reporting decreased. This trend may have been due to the decrease in social interactions due to pandemic-related social restrictions, and, if this is the case, highlights further the social issues associated with increases in race and ethnicity-based discrimination with the pandemic.

In this study we found 16% of international students in Australia at the time of the pandemic reported that ‘they ran out of food and could not afford to buy more’. This was twice the proportion who reported such issues pre-pandemic and this issue led to universities and other organizations setting up foodbanks for international students across the country [41]. The causes of these food security issues are not difficult to discern. At the start of the pandemic international students and other temporary visa holders in Australia were ineligible to apply for federal Government financial assistance, yet many experienced loss of casual work and income that they depended upon [42]. Such decisions, which appear to lack consideration of the fact that education was Australia’s third largest export prior to the pandemic, may lead to a downturn in students returning to Australia [41].

Increasing rates of domestic violence during the pandemic have been another serious concern [43]. Although research in this area is emerging, studies suggest that the pandemic has both exacerbated pre-existing violence and been a catalyst for new violence in relationships [43]. Our study found that, of the students who reported being afraid of a partner during the pandemic, most reported that their level of fear had increased across this period. This was particularly the case for international students. There are several possible explanations for this; international students may have experienced higher levels of fear due to their isolation from social supports that may have been available pre-pandemic; or it may be, as our findings suggest, that international students experienced more financial stressors which can exacerbate existing issues in relationships [43]. In any case, the findings speak to the need for increased support for international students experiencing fear of their partner.

At the time of the pandemic the issues faced by international students were reported in numerous media opinion pieces [41, 44], and a limited number of quantitative studies focusing on students from specific countries [35] or on specific issues, such as food security for temporary visa holders [42]. No previous study has included sufficient participant numbers or follow-up period to enable examination of the relationships between these issues between international and local students. When considering the issue of worsening mental health during the COVID-19 pandemic for the general university student population we can compare our study to the studies that have had a pre-pandemic comparison [19, 20]. Ours is the largest study of its type reporting such a comparison and the percentage increases observed in our study were comparable to those found in previous studies [19, 20], emphasising the generalisability of our results.

### Strengths and limitations

A primary strength of this study is that we assessed pre-COVID-19 pandemic data, and hence, changes associated with the COVID-19 pandemic can be quantified. Another strength is that it is the largest study of university students with a pre-pandemic comparison available, and therefore has provided the opportunity to compare outcomes for sub-populations of students, such as local versus international students. One of the limitations of this study is that pandemic outcomes were measured at a single point in time and may not be representative of sustained health and wellbeing issues. Hence the long-term psychosocial impacts of the COVID-19 pandemic require further assessment. An issue within any study reporting prevalence is representativeness. We were able to establish that our sample was broadly representative of the university student population from which it was drawn by making comparison with demographic characteristics available to us from the source population. We further addressed the issue of representativeness by examining loss to follow-up and missing data using an inverse proportional weighting approach. Hence, we feel that this study presents a valid estimate of issues experienced by university students during the pandemic. It can also be noted that our pre-pandemic levels of depression are similar to those reported in a systematic review of studies of student mental health issues in the pre-COVID-19 pandemic period [6].

### Implications

There are several important implications of this research. Our prior baseline survey of the mental health and wellbeing of this cohort in pre-pandemic times highlighted a need for strategies to promote, prevent and manage the mental health and wellbeing of university students [25]. This current study confirms that universities must also work to manage the existing inequities across student groups to prevent even more negative impacts when there is a shock to the system (i.e., a pandemic). Social and financial support is important, along with psychological strategies, to manage depressed mood and anxiety. Health practitioners and faculty alike need to be alert to psychosocial stressors amongst university students and especially in times of heightened social isolation brought on by a pandemic.

Implementing frameworks and guidelines to support prevention of mental health issues at universities needs to be prioritized [45–47]. While many institutions are already developing strategies to improve student mental health, a major limitation is the lack of research as to the effectiveness of many of the initiatives [48]. Even more pressing is the issue of limited research regarding interventions to best assist international students, who, as this study indicates, are a group experiencing mental health inequities.

A prevalent issue was a relative lack of social support experienced by international students. This was notable in the pre-pandemic phase and then exacerbated with the pandemic. The association between social support, social capital and mental health has been well studied [37]. Challenges remain in identifying effective methods to build social support and social capital [49]. Studies with international students have pointed to the importance of establishing student groups on-campus and offering group-based social skills training [50]. However, methodological limitations of these studies mean they lack sufficient strength of evidence for recommendations to be made [50, 51]. There is a pressing need to identify effective ways to build international student social support.

## Conclusion

This study demonstrated that university student mental health substantially deteriorated during the COVID-19 pandemic. Of note was the substantial worsening of international students’ mental health, social support, and financial security. Whilst these issues were exacerbated by the pandemic, all issues were prevalent prior to the pandemic and may well continue post-pandemic. Identifying and implementing adequate preventative interventions, such as building social capital programs at universities and in the community, is an imperative. However, further on-the-ground knowledge is still required to identify effective interventions in the current climate and into the future.

## Supplementary Information

**Additional file 1.** Additional analysis and supplementary tables.

## Data Availability

The datasets generated and/or analysed during the current study are not publicly available due ethical and legal constraints and the sensitive nature of this data but are available from the corresponding author on reasonable request.
