# Supplementary material for: Changes in mental health across the COVID-19 pandemic for local and international university students in Australia: a cohort study
Source: BMC Psychol. 2023 Feb 28;11:55. doi: 10.1186/s40359-023-01075-9 (PMC9973240; doi:10.1186/s40359-023-01075-9)
Supplement: Supplementary file 1 — Additional file 1. Additional analysis and supplementary tables. [file 40359_2023_1075_MOESM1_ESM.docx]

# Supplementary files

## Analysis

To investigate the effect of loss to follow-up between Wave 1 and Wave 2 on the prevalence measure estimates inverse proportional weighting (IPW) was applied (1). For an IPW approach a logistic regression model was developed to predict the loss to follow up. Factors from the pre-pandemic (Wave 1) were retained as predictors of loss if they were statistically significant predictors of loss and had less than 5% missing data. Numerical factors were transformed when non-normally distributed. The factors that were retained included age (reciprocal), gender, age, citizenship status, course level, hours spent on campus pre-COVID-19 (reciprocal), agreement to the data sharing of marks in the Wave 1 survey. Some of these factors were predictive of loss to follow up but are not recorded in this study, as they were not key demographic factors. The inverse of the probability of participating in the pandemic study was utilized as a weighting factor. Hence groups of participants with a low probability of participating in the pandemic were given higher weightings to account for those lost (i.e., male undergraduate international students). These weights were then applied to all prevalence measures (results not shown) and measures of association (Supplementary Table 1).

In a post-hoc analysis the inverse proportional weighting approach described above was also applied to deal with the issue of missing data at baseline (PHQ-2; GAD-2, MOS-SSS-6; being unable to afford food; percentage experiencing discrimination based on race, ethnicity and gender). In this analysis the predictors of missing outcome data at baseline were gender, international and local student status, field of study, full-time or part-time enrolment in the university course, agreement to the data sharing of marks in the Wave 1 survey. As detailed in the paragraph above participants with a higher probability of having missing data at baseline were given higher weightings to account for those lost. These weights were then applied to measures of association (Supplementary Table 1).

Stata 16 was utilized for all analyses.

## Results

**Supplementary Table 1 Weighted Local and international student change in mental health issues and other stressors from pre-pandemic to during the pandemic**

| **Mental health issues and other stressors** | **Analyses adjusted for loss to follow up at Wave 2** | | **Analyses adjusted for missing data at baseline for the outcome variable** | | |
| --- | --- | --- | --- | --- | --- |
|  | **Weighted adjusted odds of outcome for international students compared to local student^a^ (95%CI)**  **(n=4407)** | **p value for the association** | **Weighted adjusted odds of outcome for international students compared to local student^a^ (95%CI)**  **(n=4407)** | | **p value for the association** |
| **Percentage with probable major depression (PHQ2 ≥ 3) (95%CI)** | 1.43 (1.23, 1.68) | p<0001 | 1.39 (1.12, 1.73) | | 0.003 |
| **Percentage with probable anxiety (GAD2 ≥ 3) (95%CI)** | 1.11 (0.95, 1.29) | p=0.205 | 1.03 (0.83, 1.28) | | 0.781 |
| **Percentage reporting low social support (MOS-SSS-6 <18) (95%CI)** | 2.70 (2.27, 3.19) | p<0.001 | 2.68 (2.24, 3.21) | | p<0.001 |
| **Percentage unable to afford food (95%CI)** | 5.26 (4.00, 6.91) | p<0.001 | 5.59 (2.95, 7.13) | | p<0.001 |
| **Percentage experiencing discrimination (95%CI)** |  |  |  | |  |
| Based on race | 2.29 (1.86, 2.82) | p<0.0001 | 2.21 (1.79, 2.72) | | p<0.001 |
| Based on ethnicity | 1.58 (1.27, 1.96) | p<0.0001 | 1.58 (1.27, 1.96) | | p<0.001 |
| Based on gender | 0.51 (0.41, 0.64) | p<0.0001 | 0.50 (0.40, 0.63) | | p<0.001 |
| **Percentage reporting fear of partner during the pandemic** | *3.46 (2.26, 5.13)* | *p<0.001* | *-* | |  |
| **Percentage reporting increased fear of partner during the pandemic** | *1.76 (0.54, 5.75)* | *p=0.350* | *-* |  | |

PHQ2: Patient Health Questionnaire – 2 item, GAD-2: Generalised anxiety scale – 2 item. MOS-SSS-6: MOS Social Support Survey CI: Confidence interval

^a^Adjusted for age and gender and the outcome factor at baseline

**Supplementary Table 2. Change of depression, anxiety and social support across local and international students pre-pandemic (Wave 2) during the pandemic (Wave 2)**

|  | **All students**  **(n=4407)** | | **Local students**  **(3162)** | | **International students**  **(1,245)** | | **Mean change in outcome for international students compared to local students** |
| --- | --- | --- | --- | --- | --- | --- | --- |
|  | **Wave 1** | **Wave 2** | **Wave 1** | **Wave 2** | **Wave 1** | **Wave 2** |  |
| **Mean depression (PHQ-)2 (95%CI)** | 1.7 (1.7, 1.7) | 2.4 (2.3, 2.4) | 1.6 (1.6, 1.7) | 2.3(2.2, 2.3) | 1.9 (1.8, 1.9) | 2.6 (2.5, 2.7) | 0.25 (0.14, 0.36) p<0001 |
| *Missing* | *456* | *66* | *311* | *38* | *145* | *28* | *529 - 535* |
| **Mean anxiety (GAD-2)(95%CI)** | 2.2 (2.1, 2.2) | 2.8 (2.8, 2.9) | 2.2 (2.1, 2.3) | 2.8 (2.7, 2.9) | 2.0 (1.9, 2.1) | 2.8 (2.7, 2.9) | 0.10 (-0.01, 0.21) p=0.08 |
| *Missing* | *527* | *55* | *356* | *28* | *171* | *27* | *591* |
| **Mean MOS-SSS-6 (95%CI)** | 17.1 (16.9, 17.3) | 17.0 (16.8, 17.2) | 18.2 (18.0, 18.4) | 18.3 (18.1, 18.4) | 14.1 (13.7, 14.5) | 13.8 (13.4, 13.2) | -2.24 (-1.89, 2.59) p<0.001 |
| *Missing* | *527* | *55* | *356* | *28* | *171* | *27* | *591* |

PHQ2: Patient Health Questionnaire – 2 item, GAD-2: Generalised anxiety scale – 2 item. MOS-SSS-6: MOS Social Support Survey CI: Confidence interval

**Supplementary Table 3. Mental health and other outcomes for local and international students on the basis of country of living**

|  | **Odds of outcome for those living in Australia compared to overseas (95%CI)** | | **Interaction p value between local and international students and country of living** | **Weighted interaction p value between local and international students and country of living** |
| --- | --- | --- | --- | --- |
|  | **Local students**  **(3162)** | **International students**  **(1,245)** |  |  |
| **Probable major depression (PHQ2 ≥ 3) (95%CI)** | 0.44 (0.20, 0.96) p=0.04 | 1.18 (0.82, 1.68) p=0.37 | p=0.041 | p=0.052 |
| *Missing* | *351* | *179* |  |  |
| **Probable anxiety (GAD-2) (95%CI)** | 0.45 (0.19, 1.05), p=0.7 | 1.09 (0.76, 1.57), p=63 | p=0.046 | p=0.099 |
| *Missing* | *359* | *185* |  |  |
| **Reporting social support never/rarely/some of the time as compared to most/all of the time (MOS-SSS-6 <18) (95%CI)** | 0.31 (0.13, 0.75) p=0.01 | 1.26 (0.88, 1.82) p=0.21 | p=0.010 | p=0.002 |
| *Missing* | *388* | *205* |  |  |
| **Unable to afford food (95%CI)** | unavailable | 2.68 (1.46, 4.89) p=0.001 | Insufficient data | Insufficient data |
| *Missing* | 130 | 150 |  |  |
| **Experiencing discrimination (95%CI)** |  |  |  |  |
| Based on race | 1.03 (0.30, 3.55) p=0.96 | 1.28 (0.86, 1.90) p=0.23 | p=0.595 | 0.992 |
|  | 413 | 214 |  |  |
| Based on ethnicity | 1.06 (0.33, 3.44) p=0.92 | 1.13 (0.74, 1.74) p=0.57 | p=0.970 | p=0.959 |
| *Missing* | 413 | 214 |  |  |
| **Reporting fear of partner during the pandemic** | 0.42 (0.06, 3.25) p=0.407 | 0.78 (0.35, 1.76), p=56 | *p=0.637* | *p=0.756* |
| **Reporting increased fear of partner during the pandemic** | Insufficient data | Insufficient data | Insufficient data | Insufficient data |

PHQ2: Patient Health Questionnaire – 2 item, GAD-2: Generalised anxiety scale – 2 item. MOS-SSS-6: MOS Social Support Survey CI: Confidence interval

^a^Adjusted for age and gender and the outcome factor at baseline

**Supplementary Table 4. Self-reported impact of COVID-19 on students at Wave 2 (n=4407)**

| **Impact of COVID-19 pandemic on…** | **Worse than before n (%)** | **Unchanged n (%)** | **Better than before n (%)** | ***Missing n (%)*** | ***Not applicable n (%)*** |
| --- | --- | --- | --- | --- | --- |
| ***Health and wellbeing*** |  |  |  |  |  |
| My mental health | 3,041 (70.8) | 846 (19.7) | 407 (9.5) | *95 (2.2)* | *18 (0.4)* |
| My physical health | 2,333 (54.2) | 1,113 (25.9) | 855 (19.9) | *95 (2.2)* | *11 (0.3)* |
| Homesickness | 1,535 (47.0) | 1,534 (46.9) | 200 (6.1) | *94 (2.1)* | *1,044 (23.7)* |
| Access to general health service | 1,396 (34.0) | 2,478 (60.4) | 232 (5.7) | *94 (2.1)* | *207 (4.7)* |
| Access to mental health service | 917 (24.2) | 2,558 (67.5) | 316 (8.3) | *94 (2.1)* | *522 (11.8)* |
| Racial discrimination | 666 (24.0) | 2,041 (73.5) | 69 (2.5) | *94 (2.1)* | *1537 (34.9)* |
| **Study** |  |  |  |  |  |
| Connection to the University | 3,441 (80.1) | 707 (16.5) | 148 (3.5) | *105 (2.4)* | *6 (0.1)* |
| Coping with study | 3,008 (70.0) | 693 (16.1) | 594 (13.8) | *94 (2.1)* | *18 (0.4)* |
| Finding a suitable place to study | 2,606 (61.9) | 1,430 (34.0) | 172 (4.1) | *110 (2.5)* | *89 (2.0)* |
| ***Connections with others*** |  |  |  |  |  |
| Connection to university peers | 3,540 (82.5) | 530 (12.4) | 220 (5.1) | *106 (2.4)* | *11 (0.3)* |
| Connection with friends | 3,255 (75.8) | 740 (17.2) | 297 (6.9) | *105 (2.4)* | *10 (0.2)* |
| Relationship with my partner | 585 (22.9) | 1,261 (49.4) | 708 (27.7) | *106 (2.4)* | *1747 (39.6)* |
| Relationship with my family | 904 (21.1) | 2,203 (51.4) | 1,176 (27.5) | *105 (2.4)* | *19 (0.4)* |
| ***Finance and housing*** |  |  |  |  |  |
| Confidence in future job prospects | 3,062 (72.0) | 967 (22.7) | 223 (5.2) | *94 (2.1)* | *61 (1.4)* |
| Access to paid work | 2,349 (60.0) | 1,291 (33.0) | 273 (7.0) | *110 (2.5)* | *384 (8.7)* |
| Financial position | 1,833 (43.4) | 1,488 (35.2) | 902 (21.4) | *110 (2.5)* | *74 (1.7)* |
| Finding suitable accommodation | 674 (19.4) | 2,630 (75.8) | 165 (4.8) | *110 (2.5)* | *828 (18.8)* |

**Reference**

1. Howe CJ, Cole SR, Lau B, Napravnik S, Eron JJ, Jr. Selection Bias Due to Loss to Follow Up in Cohort Studies. Epidemiology (Cambridge, Mass). 2016;27(1):91-7.
